# Exploring the Role of Genetic and Environmental Features in Colorectal Cancer Development: An Agent-Based Approach

**DOI:** 10.3390/e26110923

**Published:** 2024-10-30

**Authors:** Marco Ledda, Alessandro Pluchino, Marco Ragusa

**Affiliations:** 1 Dipartimento di Fisica e Astronomia Ettore Majorana, Università di Catania, 95123 Catania, Italy; alessandro.pluchino@ct.infn.it; 2 INFN Sezione di Catania, 95123 Catania, Italy; 3 Dipartimento di Scienze Biomediche e Biotecnologiche, Sezione di Biologia e Genetica, Università di Catania, 95123 Catania, Italy; mragusa@unict.it

**Keywords:** agent-based models, colorectal cancer (CRC), computational biology

## Abstract

The complexity of issues in cancer research has led to the introduction of powerful computational tools to help experimental in vivo and in vitro methods. These tools, which typically focus on studying cell behavior and dynamic cell populations, range from systems of differential equations that are solved numerically to lattice models and agent-based simulations. In particular, agent-based models (ABMs) are increasingly used due to their ability to incorporate multi-scale features, ranging from the individual to the population level. This approach allows for the combination of statistically aggregated assumptions with individual heterogeneity. In this work, we present an ABM that simulates tumor progression in a colonic crypt, to provide an experimental in silico environment for testing results achieved in traditional laboratory research and developing alternative scenarios of tumor development. The model also allows some speculations about causal relationships in biologically inspired systems.

## 1. Introduction

The construction of a generalized model that simulates tumor development in a given tissue of a human organism is a challenging task, encompassing various analytical and computational difficulties. These difficulties arise from issues related to data availability, modeling, and organization, as well as to the complexity and irregularity of genomic and histological features observed in different tumors [1]. Consequently, researchers have attempted to analyze various tumors as quasi-isolated phenomena, considering their specific cellular functions, genotypes, and phenotypes [2].

Among all cancer types, colorectal cancer (CRC) stands out as the only one for which a clear progression model exists. This model describes the progression from physiological–neoplastic epithelium to metastasis in terms of the activation and suppression of a series of specific genes [3], based on statistical data on genetic mutation frequencies and chromosome deletions. Subsequent discoveries identified three genetic agents present in every human tumor: oncogenes (e.g., KRAS), tumor suppressors (e.g., APC and TP53), and stability genes [4].

CRC progression is also characterized by other important genomic features, such as chromosomal instability (CIN), microsatellite instability (MSI), DNA mismatch repair deficiency (MMR), and DNA methylation; it is a well-studied epigenetic cause of tumor development [5]. The oncogenic multi-step process begins with the deregulation of the β-catenin pathway due to an APC mutation, followed by additional somatic mutations that enhance cell proliferation (KRAS mutation) and apoptosis resistance (TP53 mutation) [4]. These genomic deregulations occur with varying frequencies in the ideal timeline of CRC development [6].

Efforts to describe, classify, and predict the origins and evolution of CRC have led to the development of various experimental tools, including in vivo, in vitro, and in silico models [7,8,9,10,11,12,13,14]. Each approach has its own strengths and limitations. In vivo animal models provide strong evidence about molecular and genetic alterations or how therapies affect tumor development. However, they may not always accurately represent human responses due to genetic complexity and differences in lifestyle [15,16]. In vitro models allow direct observation and control at the cellular level; however, standardization of cellular features is required for reliable results. Even though there has been an increase in the bioengineering of 2D and 3D tissue models for evaluating drug efficacy, some aspects, e.g., colonic flora, remain an issue [8,15,17,18]. Recently, in silico models have offered several opportunities for a different approach.

In the last two decades, advancements in computational power have facilitated the development of various computational methods, such as lattice models, off-lattice models, and cell-based models, which simulate key characteristics of cancer and processes involved with it, including hypoxia, angiogenesis, drug delivery, cancer stem cell origination, immune system interactions, the invasion of adjacent tissues, and metastasis [19]. For instance, the phenomenon of *anoikis* (a particular form of programmed cell death, triggered when a tissue cell detaches from the extracellular matrix) can be effectively simulated using lattice models, where cells have their own defined space. When inter-cellular adhesion is weakened by mutations and the pressure between cells exceeds a specific threshold, related to a minimal distance from these cells to the basement membrane, detached cells undergo cell death [20].

Among these methodologies, an interesting approach is represented by agent-based models (ABMs). By simulating individual agents, interacting in virtual environments through simple dynamical rules, ABMs enable the study of emergent phenomena in complex systems at different spatial scales. If opportunely calibrated with real data, these models can produce results coherent with observed stylized facts and can be used for simulating different counterfactual scenarios. ABMs have demonstrated their effectiveness in several contexts, from the socioeconomic level to ecological and biological ones [21,22,23,24,25,26,27,28,29]. In the biological context, which is what we are interested in here, ABMs have been applied to cancer development. For example, mono-crypt models have been extended to incorporate proliferation in multiple crypts, attempting to simulate the dynamics of an entire portion of the colon tissue [30]. Other approaches involve comparing cell-based spatial models, incorporating cell–cell adhesion and proliferation changes in response to Wnt signals, with stochastic one-dimensional models predicting the evolution of monoclonal conversion [31]. Genetic-focused models study the dynamics of cell populations within the crypt, considering them as evolving populations due to changes in genotype frequencies. The output of these models can be compared with that of genomic analysis and with the output of agent-based models in which sub-clones are generated as a result of driver mutations [32].

Building upon these existing models and the theoretical work of Fearon–Vogelstein [3], our objective is to develop an agent-based model that simulates cell dynamics in the colonic crypt, as an experimental environment for testing various scenarios related to CRC development. Furthermore, our approach aims to integrate genetic triggers, spatial and population constraints, as well as interactions with other species (such as immune system-like cells). The model is designed to represent a biological evolutionary system, considering cells in the crypt microenvironment as individuals belonging to different populations and types [33], subjected to evolutionary mechanisms (there is still an important ongoing debate about necessary and sufficient conditions to assess whether it is possible to talk about cellular species or cellular types [34]. However, in this work, the terminology will be used since the simulated cells are conceived as general asexual organisms, missing all the complex molecular, genetic, and morphological characteristics that make them so difficult to classify in real organisms). This perspective is based on the idea that biological systems can be viewed as hierarchically nested networks, where each network represents a particular level. Following this perspective, cells, as independent agents, could be considered as networks composed of molecular elements at a lower level (such as DNA and the various RNA), but also, at a higher level, as nodes of a cellular population network, working together to carry out tissue and organ functions [35].

The remainder of the paper is organized as follows: Section 2 provides a brief description of the crypt dynamics from a biological perspective and outlines the features of the proposed model. In Section 3, we describe and explain the results obtained from the simulations. Section 4 discusses the implications of the results. Finally, Section 5 summarizes the work and presents future directions.

## 2. Methods

### 2.1. Biological Background

Colonic crypts represent invaginations within the colon tissue, measuring approximately 300 μm in length with a diameter of 100 μm [36]. Crypts are dynamic structures where enterocytes, which are the intestinal epithelial cells, undergo a series of coordinated movements. These enterocytes are generated by stem cells located at the base of the crypt and ascend toward the crypt mouth, ultimately reaching the lumen of the colon. During this continuous movement, enterocytes receive signaling molecules known as Wnt ligands from neighboring mesenchymal cells; this process plays a crucial role in stimulating the production of β-catenin, a protein that exhibits a descending gradient along the crypt, with higher concentrations at the base [2,37]. Enterocytes possessing elevated levels of β-catenin are referred to as transit-amplifying cells. These cells display characteristics similar to stem cells and exhibit a higher proliferation activity, thereby ensuring a constant supply of new enterocytes in the crypt. As enterocytes undergo upward migration, there is a gradual reduction in the concentration of β-catenin. This decline occurs because β-catenin is continuously targeted for degradation by a protein complex known as the destruction complex, thereby impeding its nuclear translocation. This reduction allows the cells to eventually undergo differentiation (assuming specific functions) and to eventually undergo apoptosis within a span of 3 to 5 days [2,38]. This process represents an extraordinary mechanism for safeguarding against cellular dysfunctions resulting from DNA damage or other challenges arising within the intestinal microenvironment [2].

### 2.2. The Agent-Based Model

**Figure 1:**
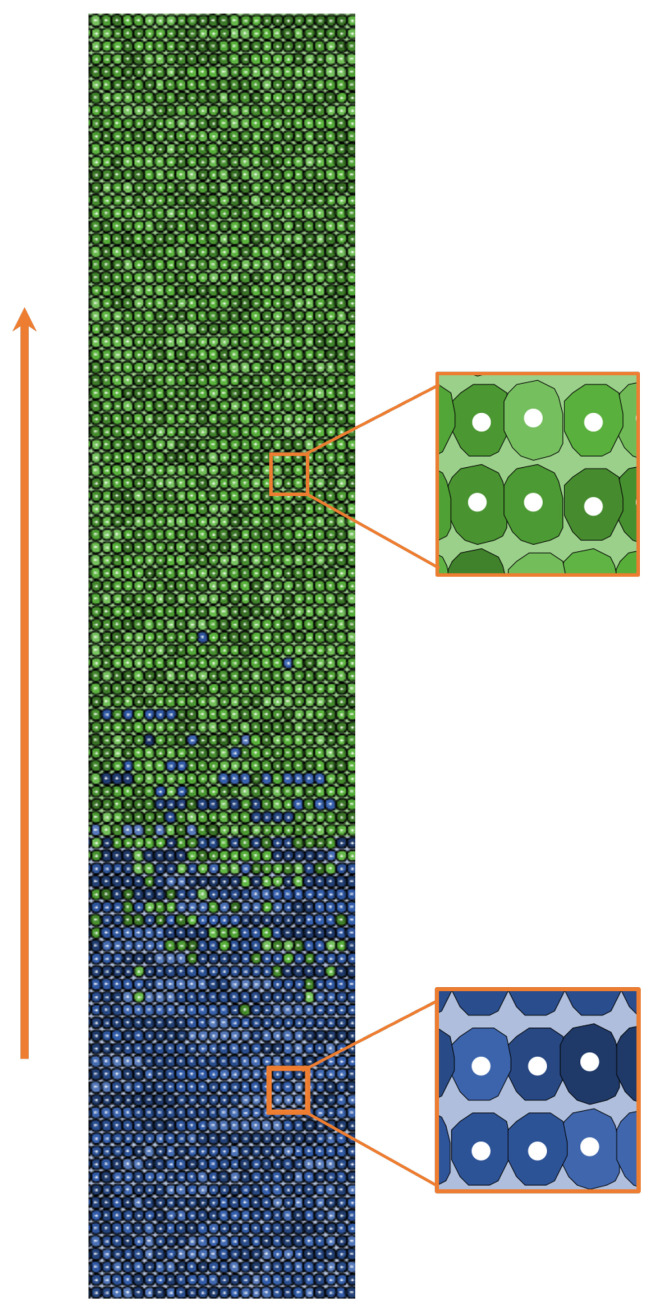
The colonic crypt simulated in the NetLogo world is represented as an unfolded cylinder (rectangle), 100 layers in height and 25 cells per layer. Cell typologies: blue cells represent *transit-amplifying* cells and green cells represent *differentiated* cells. The orange arrow indicates the direction of movement. The white dots in the center of the cells and the different green and blue shades are only aesthetic additions with no influence on cellular behavior.

To model the above-described process, we used NetLogo (version 6.2.2) [39], an interactive, fully programmable multi-platform environment specifically designed for agent-based simulations. Figure 1 shows a 2D representation of the colonic crypt in the NetLogo virtual world, arranged as if the crypt were an unfolded cylinder.

#### 2.2.1. World Structure

Our case study corresponds to the median crypt, typically consisting of 2000–2500 cells arranged in 100 layers (lines along the vertical axis) with 25 cells per layer, see Figure 1. Since each cell has a length of 3 patches and a width of 2, the World size is set at 300 × 50 patches. These patches represent the surface where cells are positioned. In reality, this surface includes stromal cells responsible for constructing the supportive extracellular matrix for enterocytes. These stromal cells are believed to transmit Wnt signals to the stem cells located at the base of the crypt [2,37]. In the crypt-world, physiological cells are distributed in a predefined proportion, with approximately 75% being *differentiated* cells and 25% being *transit-amplifying* cells, as reported in the literature (see [20,40,41,42,43] and references therein for more details). The simulated β-catenin quantity is set at 1 at the bottom of the crypt and decreases to 0 at the top, with the decrease rate arbitrarily chosen to maintain a constant proportion between transit-amplifying and differentiated cells. In the model, the epithelium goes through renewal every 3–5 days, indicating that each cell has a timeframe of 72–120 h to either reach the crypt mouth or undergo cell death [44]. Thus, we assign to physiological cells a lifespan consistent with this constraint, as shown in Table 1. At each simulated time step (1 tick), corresponding to 1 h of real-time, the physiological cells move toward the top of the crypt of one layer. By assigning an age equal to 0 for cells in the first line and equal to 100 for the cells in the last line that did not die during migration, the maximum migration time closely aligns with reality, which is between 100 and 120 h [45,46]. Considering the crypt as an unfolded cylinder, the model adopts horizontal periodic boundary conditions, such that the first cell starting from the left side of the crypt is adjacent to the first cell starting from the right side. When cells reach the topmost cell line, they exit the simulation and are no longer considered in the model dynamics.

#### 2.2.2. Agents Behavior

NetLogo agents represent cells. They are divided into two physiological typologies *(transit-amplifying* cells and *differentiated* cells, already introduced) and three neoplastic typologies *(pre-adenoma*, *adenoma*, and *tumoral* cells), which arise only when a genetic trigger occurs. The list of values for the relevant dynamic variables of cells is presented in Table 1 and includes both physiological and neoplastic typologies. Further details about APC, KRAS, and TP53 genes, which appear in the assignment of the values, will be addressed in the next subsection and are presented in Table 2.

**Figure 2:**
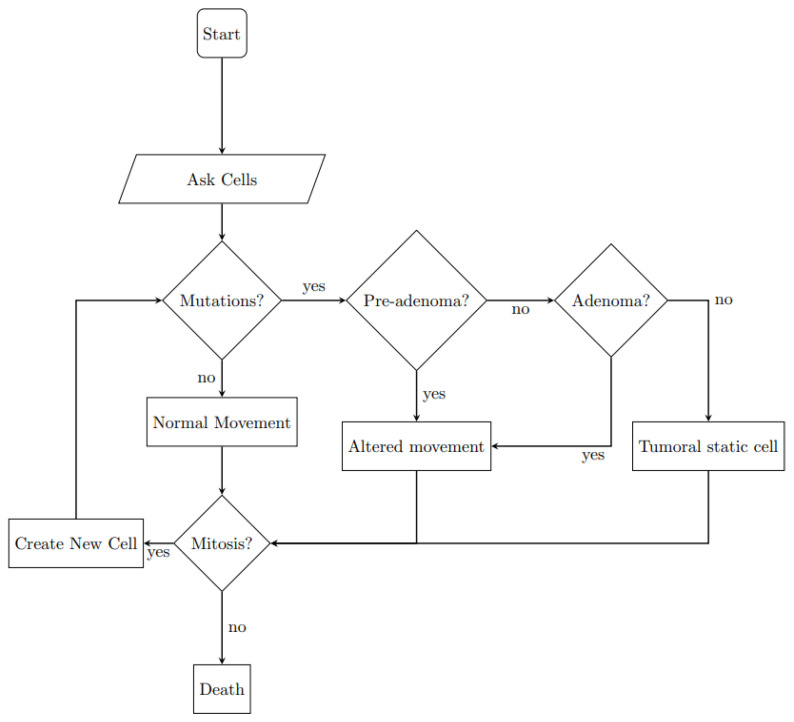
Flowchart of the basic cell behavioral algorithm.

Each individual cell carries out its activities autonomously while occupying a specific position (patch) within the World. In Figure 2 the algorithm followed by each cell is reported as a flowchart. It includes normal movements and replications, addressed below, and the possibility of mutation, which will be addressed in the next subsection.

**Figure 3:**
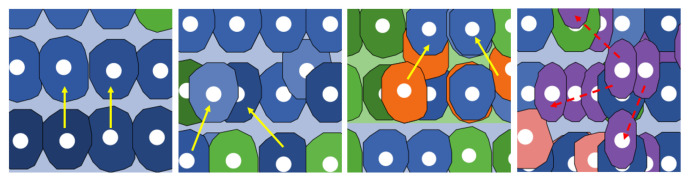
Cell movement based on cell type: *transit-amplifying* cells are represented by blue cells, *differentiated* cells by green cells, *adenoma* cells by orange cells, and *tumoral* cells by violet cells. Arrows indicate the degree of angular freedom during movement and mitosis. In the last panel, the arrows are dashed red lines because *tumoral* cells can only enter mitosis in the allowed direction, but they can no longer move. The arrows depicted here are a visual representation—within the crypt—of the direction of movements exposed in Table 1.

The movement and replication (mitosis) of cells are influenced by their interactions with neighboring cells. This modeling approach is chosen to preserve the individual heterogeneity of cell behavior while capturing population-level characteristics such as the integrity of the entire epithelium of the crypt, which remains unaltered in its structure. In the physiological state, cells have a single degree of freedom for movement. They are allowed to advance one patch forward only if the patch ahead of them is unoccupied, as illustrated in the first panel on the left of Figure 3. This movement mechanism ensures a continuous flow of cells from the bottom to the top of the crypt.

Since every cell can have a different lifespan due to random events, such as being killed by other cells or having reached the maximum lifetime, some of them leave empty spaces within the cell line. To address this issue, all cell types are capable of entering mitosis to fill these gaps. When an empty space occurs, a neighboring cell, from either the right or the left, randomly undergoes mitosis activity to occupy the vacant position. Another challenge arises when a cell reaches its mitosis time but patches to the left and the right are not empty. In such cases, the mitosis cell eliminates a neighboring cell with a lower number of mitosis activities or a higher lifetime. These processes align with biological plausibility, as a cell can be eliminated based on the number of times it has replicated (linked to its fitness) or its age. This allows for more fitted or younger cells to take their place, as observed in biological systems [48].

#### 2.2.3. Genetic Features and Mutation Effects

Every cell has a set of genes, see Table 2, chosen following the CRC progression model already described in the Introduction.

We adopt the one-hit hypothesis for oncogenes (*KRAS* in this model) and the two-hit hypothesis for tumor suppressor genes (*APC* and *TP53*). According to these hypotheses, a single allele mutation is sufficient to enhance the function of oncogenes, while both alleles of a tumor suppressor gene must be mutated for its function to be affected [4]. To incorporate the loss of heterozygosity (LOH) characteristic [49], we set the probability of point mutation in the second allele to be one order of magnitude higher than the probability of mutation in the first allele [50]. Specifically, if the probability of mutation in the first allele is $10^{-9}$, then the probability of mutation in the second allele, if the first is mutated, is $10^{-8}$. In our model, each of the three genes controls a simplified action to avoid excessive complexity.

The tumor suppressor gene *APC*, even when heterozygous, has a positive function in controlling the differentiation of cells from transit-amplifying to differentiated state. However, mutation of both alleles in *APC* disrupts the regulation of β-catenin, preventing cells from differentiating. This can lead to the migration of transit-amplifying cells into regions of the crypt where only differentiated cells should be present. Additionally, we introduce a slight deviation in the forward movement of cells to simulate a slower rate of migration, which may contribute to cell accumulation after mitosis.

Regarding the *KRAS* gene, mutation of one allele increases the mean proliferation time [51]; refer to Table 1 for the directions of movement and replication.

The wild-type version of *TP53*, known as the *genome Gatekeeper* [2,52], plays a crucial role in cell regulation. In its wild-type homozygote form, it ensures that cells with significant DNA errors undergo apoptosis. In this model, the process develops as follows: each of the 50 *regulation genes* comprises a list (structured similarly to the driver genes). When a mutation occurs, the values in the list change from 0 to 1. Consequently, the total sum of the list can only increase. If the sum exceeds a certain threshold and *TP53* is a wild-type homozygote, the cell dies. However, when both alleles of *TP53* are mutated, it increases the probability of mutation in all other genes. Specifically, the probability exponent decreases by 0.5 units for each mitosis until cellular death. The precise quantitative analysis for this phenomenon is not available in the literature, so we chose a decrease rate that is neither too pronounced nor insufficient to realistically simulate DNA deregulations. It is worth noting that *TP53* is described in the literature as a tumor suppressor gene involved in crucial aspects of neoplastic and metastatic processes, such as proliferation, resistance to apoptosis, and migration [2,53]. Therefore, it seemed appropriate to simulate the various functions of *TP53* by augmenting the overall probability of mutation in other genes, even though to an arbitrary extent.

Although there is no specific temporal order for the occurrence of mutated genes in neoplastic typologies, various combinations of mutated genes lead to different types of neoplastic variants. When a cell acquires the *pre-adenoma* typology, instead of moving in a straight line, it inclines randomly to the right or left, as depicted in the second panel from the left in Figure 3. If, in addition to the *APC* mutation, the *KRAS* gene also undergoes mutation, the cell transitions to the *adenoma* typology. Cells with the *adenoma* typology exhibit the same movement pattern as *pre-adenoma* cells but proliferate at a faster rate, as shown in the third panel; Figure 3. The final phenotypic change occurs with the addition of the *TP53* mutation, resulting in the *tumoral* typology. In this state, cells are unable to move around but can proliferate in all positions rather than just adjacent spaces, as illustrated in the fourth panel of Figure 3.

#### 2.2.4. Micro-Environmental Features

Genetic factors are well-known triggers of cancer evolution in many types of cancer, and despite the fact that representing these factors sometimes can be challenging, modeling the influence of environmental factors poses an even greater challenge.

Our current understanding of the *tumor microenvironment* (TME) highlights the complexity of elements present in our body from the early stages of tumor development to metastasis [54]. These elements play roles in either promoting or inhibiting tumor progression. Simulating every specific molecular interaction within the TME would be impractical and inconsistent with the intended scope of the model. Therefore, we chose to represent immune system cells as a combination of some functions belonging to natural killer (NK) cells and macrophages (M1 and M2) to account for the complexity of the TME [55]. In particular, the function of NK cells is to patrol the body, constantly surveying for abnormal cells, and identifying and killing cells that have become cancerous. Moreover, the M1 and M2 functions involve eliminating cellular debris from the body.

Another important environmental feature of the crypt structure is the number of cells present in a patch. Physiological cell disposition is rigid and is set up to one cell for every spot (the spot dimension is 3×2 patches). Thus, when a cell with typology belonging to neoplastic variants moves or proliferates in the spot of another cell, the threshold counter starts. In real crypts, over-proliferating cells tend to create hypoxic niches, where cells situated at a certain distance from the nearest blood vessel are subjected to a lack of oxygen eventually dying [56]. To capture this phenomenon, we incorporate a hypoxia threshold and its effects on cell survival. The interaction between epithelial cells and environmental features follows specific rules. A cell count is performed in each patch, and when a threshold is reached, immune system cells appear and move randomly within the crypt. If these immune cells encounter a non-physiological cell, they move toward it and eliminate it, mimicking NK cells and macrophage interactions. The same cell count procedure also activates another threshold, which represents the maximum number of non-hypoxic cells allowed in a given spot. By incorporating these rules, we aim to capture the essential aspects of the interaction between epithelial cells and the TME while simplifying the representation of spatial dynamics and avoiding the simulation of complex molecular interactions.

## 3. Results

In our model, the starting values of cellular genotypes can be set to homozygous wild-type or heterozygous (e.g., [0 0] or [0 1], [1 0]). The initial probability distribution for these genotypes can be adjusted using a slider in the model interface, ranging from 0 to 1. A value of 0 indicates that all cells start with a homozygous wild-type genotype, while a value of 1 means that all cells start with a heterozygous genotype. This feature is designed to account for the stem niche hypothesis, which suggests that mutations can also occur within stem cells, allowing stem cell clones to invade the entire crypt [37]. Although stem cells are not explicitly included in the model dynamics, this characteristic preserves the possibility of stem cell involvement, ensuring that if a stem cell mutation occurs, it would be inherited by the daughter transit-amplifying cells. To observe changes in the dynamics of cell population growth, the rate of cell death due to physiological and environmental causes (e.g., immune system-mediated killing or overcrowding), and the rate of cell death due to aging of tumoral cells, we conducted several runs with three different stop conditions: (*a*) one year has passed; (*b*) the proportion of transit-amplifying and differentiated cells is inverted; (*c*) the crypt population reaches 10,000 individuals (These stop conditions are arbitrary and not derived from the literature. The reasons behind them are as follows: (a) given the model characteristics, one year (365 ticks) is sufficient to observe the effects of the programmed behavior; (b) it is reasonable to think that if the proportion of transit-amplifying and differentiated cells is reversed, the crypt structure would be compromised, thus justifying the cessation of the simulation; (c) considering the physiological size of the population (2500 cells), reaching 10,000 cells would make the crypt resemble a real crypt completely filled with neoplastic cells.).

To analyze the effects of varying mutation probabilities, we adjusted the probability of gene mutation from $10^{-9}$ to $10^{-2}$ while keeping the other parameters constant. This range covers a wide spectrum of mutation probabilities and allows for meaningful comparisons with other models that have used similar values [11,50]. By examining seven scenarios ($10^{-9}$, $10^{-7}$, $10^{-6}$, $10^{-5}$, $10^{-4}$, $10^{-3}$, $10^{-2}$) with different mutation probabilities, we gained insights into how changes in mutation rates impact cell behavior and overall system dynamics. The other fixed parameters were as follows:

Number of immune system cells: 1/h
Number of cells that a killer cell eliminates before death: 5 cells.
Hypoxic threshold: 5 cells/patch.
Immune system threshold: 5 cells/patch.
Probability of starting genotype: *p* = 0.5.

The chosen parameter values are based on qualitative and quantitative descriptions from the literature [56,57]. Since some parameters, such as the concentration of immune system cells produced per hour and the number of *tumoral* cells that an immune system cell can kill before dying, are not known at the level of individual cells, we have set them in the model as arbitrary values that are susceptible to manipulation. Therefore, an immune system cell cannot kill more than one neoplastic cell at a time and has a lifetime expressed in the number of neoplastic cells killed. Similarly, while the real hypoxic threshold is stated at 150 μm, the exact number of epithelial cells from a hypothetical blood vessel is not necessary in the context of our model. This characteristic pertains to the real three-dimensional structure of the crypt, which is not effectively reproducible in a two-dimensional model.

**Figure 4:**
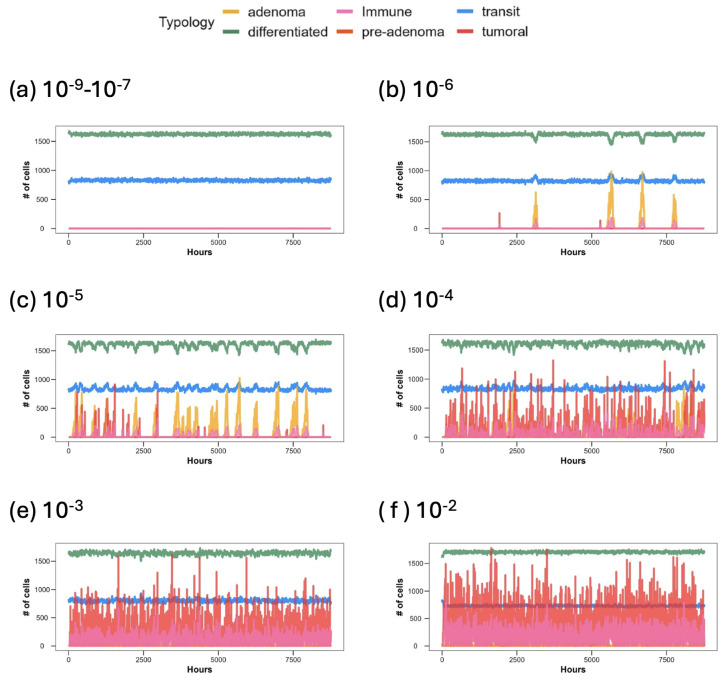
Population dynamics in the function of hours. Abbreviations are referred to as cell typologies: *transit-amplifying, differentiated, pre-adenoma, adenoma, tumoral, immune system cells*. Different panels show the size of the cell populations in crypts with a decreasing gene probability of mutation, from $10^{-9}$ (**a**) to $10^{-2}$ (**f**).

The population dynamic variations are depicted in Figure 4. Panel (a) illustrates the constant population trends of *transit-amplifying* and *differentiated* cells observed throughout the simulation period of one model year (365 ticks), with mutation probabilities ranging from $10^{-9}$ to $10^{-7}$. This panel provides an overview of the constant trend and proportions when the probability of mutation is low, indicating that mutational events are rare. When the mutation probability is within a physiological range [58], we observe the expected steady flow of cells differentiating in the correct positions, thus maintaining the imposed proportions.

In panel (b), with a mutation probability of $10^{-6}$, minor expansions of the transit-amplifying population occur, attributed to the emergence of *adenoma* and *pre-adenoma* mutations. These expansions cause a restriction of the differentiated population and a corresponding increase in transit-amplifying cells.

Panel (c) shows that cellular populations are subjected to fluctuations, alternating between growth and regression of *adenoma* and *pre-adenoma* phenotypes, resulting in a homeostatic behavior where immune system cells fight back against neoplastic variants. Additionally, it is observable that neoplastic expansions increase in frequency but not in amplitude; that is, as time passes, there are more neoplastic events but no significant increase in the size of the neoplastic populations.

Panels (d), (e), and (f) exhibit similar trends, with a significant increase in both the *tumoral* and *adenoma* populations. The system’s tendency to bounce back toward a stable physiological equilibrium is challenged by the high probability of mutation, which leads to the uncontrolled proliferation of *tumoral* phenotypes, especially in scenarios (e) and (f). Furthermore, in scenario (d), we observe an increase not only in the frequency but also in the number of *tumoral* variant cells. Scenarios with non-physiological variants show a periodic trend in the immune system cell population, closely matching the *pre-adenoma*, *adenoma*, and *tumoral* phenotypes in terms of timing. This periodicity exhibits characteristics resembling a predator–prey relationship, consistent with how the interactions between immune system cells and neoplastic cells are conceived.

**Figure 5:**
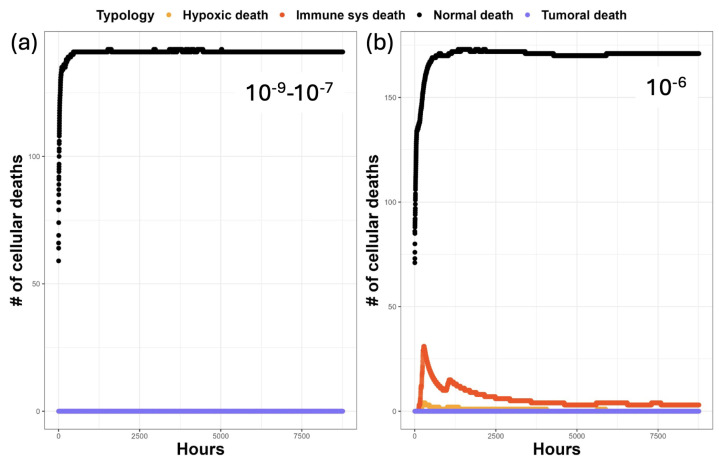
Here, various types of cell deaths occurring per hour are presented. *Physiological death* indicates the number of cells that have died due to physiological causes, such as reaching their maximum life threshold or being killed by another physiological cell to facilitate mitosis activity. *Hypoxic death* refers to cells that have died after the hypoxic threshold is reached. *Immune sys death* represents the number of cells killed by the immune system. *Tumoral death* accounts for tumoral cells that have died due to reaching the age threshold, which is set at 150 h. Panel (**a**) illustrates different scenarios, ranging from crypts with a probability of mutation as low as $10^{-9}$ up to $10^{-7}$. Panel (**b**) specifically focuses on the scenario with a probability of mutation set at $10^{-6}$.

In all the simulations conducted with mutation probabilities ranging from $10^{-9}$ to $10^{-7}$, there were no significant differences in the death rates per hour. As depicted in Figure 5a, the death rate remained relatively constant at approximately 140 cells per hour. In the scenario with a mutation probability of $10^{-6}$, significant changes in the death rates were noted. There was an increase in the rate of physiological deaths, up to 160 per hour, along with a rise in other modes of death, such as death resulting from local overcrowding (referred to as *Hypoxic death*) and cells being killed by the immune system (*Immune sys death*). An interesting observation is the absence of a significant death rate among aged tumoral cells, possibly due to the fact that very few, if any, can evade the environmental factors causing their demise, as shown in Figure 5b.

**Figure 6:**
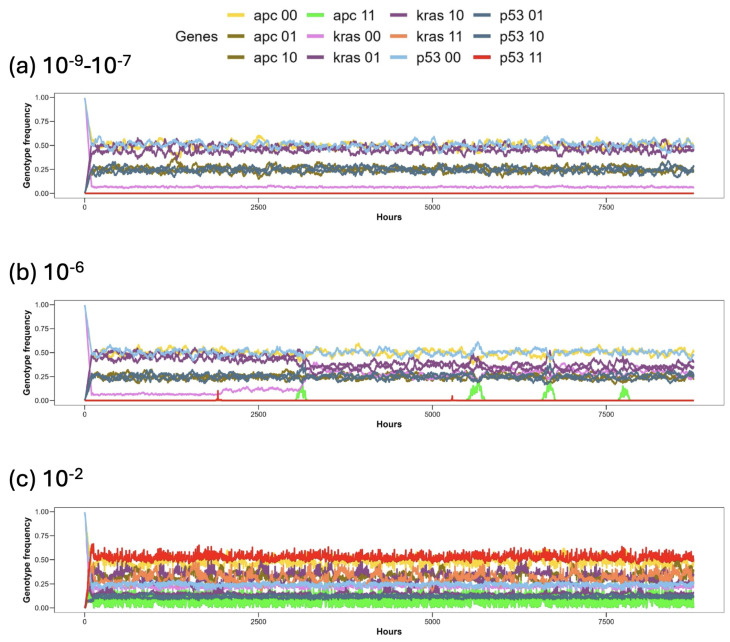
Genotype frequency of the genes in the cell population as a function of time for three different scenarios, with the following values of the mutation probability: from $10^{-9}$ to $10^{-7}$ (**a**), $10^{-6}$ (**b**), and $10^{-2}$ (**c**). The abbreviations used refer to the names of genes. For example, APC 00 represents the gene *APC* with both alleles being wild-type, APC 10 and APC 01 represent heterozygosity and APC 11 represents both alleles being mutated. The same conventions apply to the other two genes, *KRAS* and *P53*.

The analysis of genotype frequencies as a function of time revealed a correspondence between the flow of the various genotype values within the population and the dynamics of the population itself, as shown in Figure 6.

In panel (a), the genetic flow remains constant, reflecting stable population dynamics when the probability of mutation ranges from $10^{-9}$ to $10^{-7}$. During the first 120 h of total renewal of the crypt epithelium, we observe a concentration of genotypes mostly at frequencies of 0.25 and 0.50. After that period, all genotypes maintain the same frequency throughout the entire simulation. In panel (b), the frequency changes correspond to the clonal expansions observed in the population plot. Several expansions are attributed to *adenoma* mutations, with an interesting increase in the wild-type version of *KRAS* (pink line) following the first *adenoma* expansion (light green line). Panel (c) illustrates the gene flow within the crypt when the mutation probability is set to $10^{-2}$. In this scenario, the most prevalent genotypes are those with *APC* = [0 0], *KRAS* = [1 1], and *P53* = [1 1], indicating that the most common phenotype is *adenoma* with completely mutated *TP53*.

**Figure 7:**
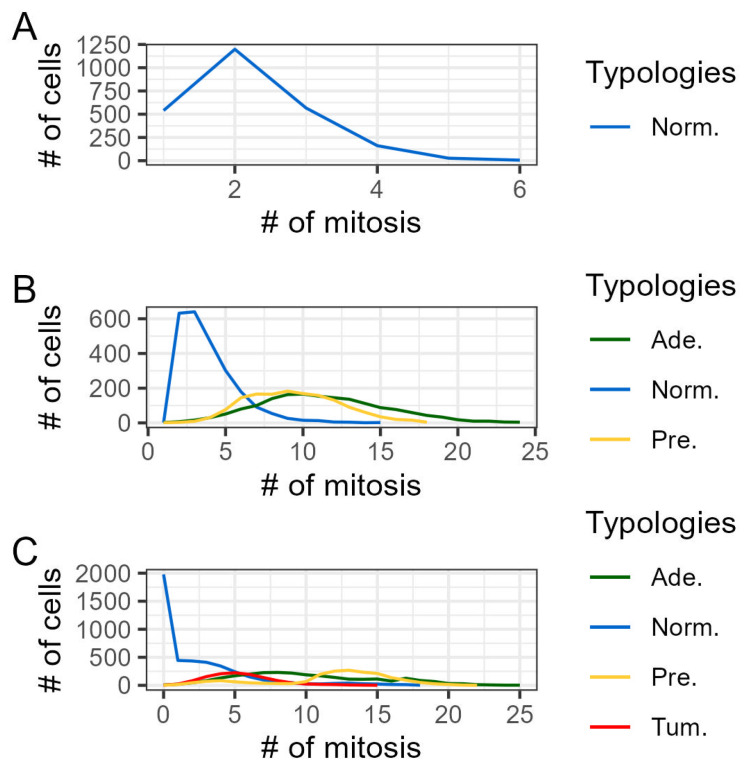
Proliferation distributions. Panel (**A**) shows the mitosis distribution in the physiological scenario. Panel (**B**) shows the scenario at $10^{-6}$ with the emergence of both, *pre-adenoma* and *adenoma* cells. Panel (**C**) shows the scenario at $10^{-2}$, where all the neoplastic typologies are present.

**Figure 8:**
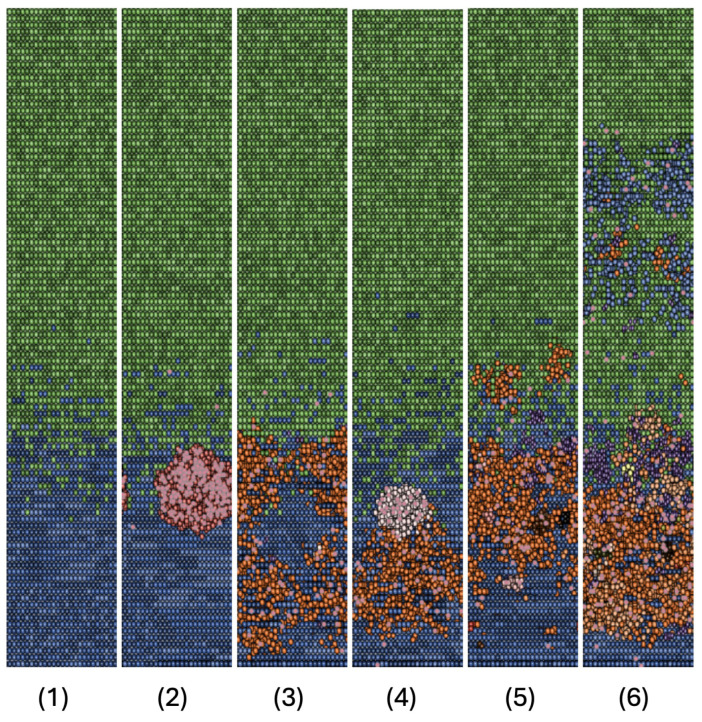
Crypt final states: starting from the left panel we have six snapshots of the nine final stages of simulated scenarios. (**1**) This scenario represents the physiological state of a healthy crypt, with *transit-amplifying* and *differentiated* cells positioned correctly along the height of the crypt and in the appropriate proportions. This physiological state remains the same even when the probability of mutation is fixed at $10^{-8}$ and $10^{-7}$. (**2**) In this scenario, changes occur and we observe a cluster of adenomatous cells being attacked by immune system cells. (**3**) This scenario represents a probability of mutation of $10^{-5}$, with no qualitative changes in the emergence and types of mutated phenotypes. (**4**) In this scenario, the probability of mutation is $10^{-4}$ and a distinction arises between two mutated phenotypes with different local displacements. (**5**) This scenario corresponds to a probability of mutation of $10^{-3}$, where there is a much more pronounced intratumoral heterogeneity (ITH), with the first mutated cells emerging at the level of differentiated cells. (**6**) Here, the probability of mutation is $10^{-2}$ and we observe a completely deregulated crypt with evident ITH. Additionally, transit-amplifying cells keep migrating toward the crypt mouth without differentiating.

Interesting results emerged concerning also the number of mitosis events for each phenotype, as seen in Figure 7. Physiological cells, for example, exhibit a skewed distribution, see panel A, while *pre-adenoma* and *adenoma* cells show a very wide distribution with a nearly shared maximum value, see panel B. In panel C, *tumoral* cells present an analogous size but a less wide distribution (a maximum of 15 mitoses per cell, compared to 22 for *pre-adenoma* and 25 for *adenoma* cells). Observing the distributions of neoplastic variants in both panels B and C, we notice that the proportions between *pre-adenoma* and *adenoma* population sizes remain almost unaltered as the mutation probability increases. These features are likely due to the sparse spatial arrangement of *pre-adenoma* and *adenoma* phenotypes, whose movement creates random mitosis spots where environmental conditions differ (see Figure 8), combined with the programmed mitosis time (see Table 1).

**Figure 9:**
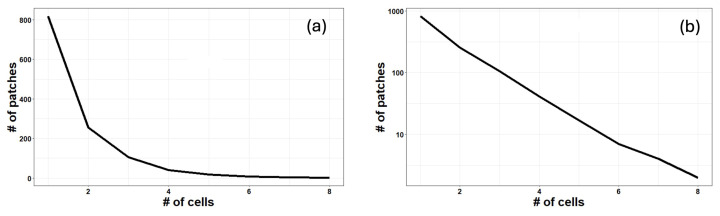
The frequency distribution of the number of cells per patch is reported for the scenario with mutation probability $10^{-2}$ at the time of maximum tumoral expansion. (**a**) lin-lin plot; (**b**) log-lin plot. It is evident the exponential behavior of the curve, decreases linearly in semi-logarithmic scale, meaning that mitosis mostly occurs in a very small number of sites within the crypt.

In Figure 9, the distribution of the number of cells per patch at the time of the maximum tumor expansion is reported in both linear (a) and semi-logarithmic (b) scales for the scenario with mutation probability $10^{-2}$. The number of patches containing a given number of cells decreases exponentially with the number of cells contained therein (the behavior is linear in the log-lin scale). This means that most mitosis events occur in a few specific sites, while the rest of the crypt’s surface shows fewer or no mitosis events per site.

A model constructed using data and assumptions from the literature should provide results that are, at least, consistent with the current state of the art. From our perspective, this consistency is evident from two aspects. Firstly, the visual representation of the cell arrangement in the crypt within the NetLogo environment aligns with the phenomenon known as *intratumoral heterogeneity* (ITH) [59], as depicted in Figure 8, 1–6. The visual aspect of the crypts reveals a significant difference between those with a probability of mutation set at $10^{-9}$, see panel (1) in Figure 8, and those with a probability of mutation set at $10^{-6}$ or higher, see panels (2–6) in Figure 8. If we compare images of crypt states with the population dynamics in the range of $10^{-9}$ to $10^{-6}$, as shown in Figure 4, we observe a phenomenon similar to a phase transition with clusters of neoplastic variants sprouting at $10^{-6}$ scenario. Furthermore, within the range of $10^{-3}$ to $10^{-2}$, we observe behavior associated with cells exhibiting chromosomal instability (CIN) [49]. This behavior is characterized by a rapid growth of the total cellular population (4000–6000 individuals) occurring within 100–150 h.

**Figure 10:**
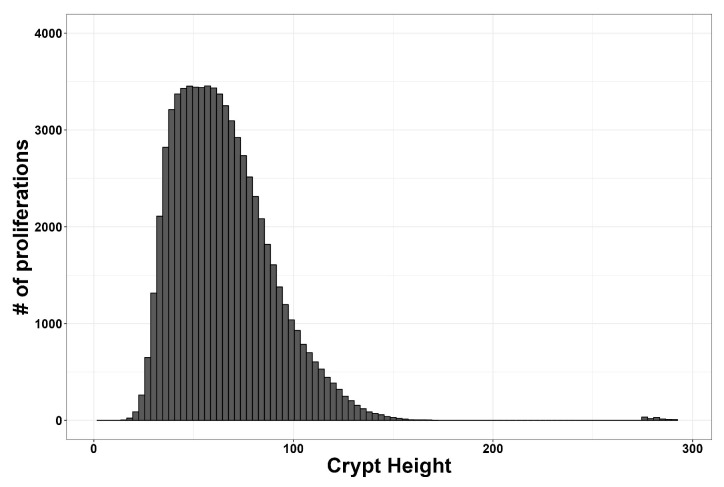
Mitosis activity based on crypt height. The height of the crypt is measured in the number of patches (0–300). The distribution shows the total number of mitosis in the physiological scenario (probability of mutation ranging from $10^{-9}$ to $10^{-7}$).

From a quantitative standpoint, the model’s design, such as proliferation events measured by crypt height, is similar to those observed in other CRC studies [41,43], as shown in Figure 10, where the majority of mitosis events occur within the transit-amplifying population.

## 4. Discussion

The purpose of developing this model was to create a tool for theoretical investigations, hypothesis generation, and experimental design related to a biological phenomenon. Modeling complex biological phenomena is inherently challenging, therefore, we would like to reflect on the variable choices, parameter ranges, and the consistency of our results with other studies.

The variables within the model are intentionally designed to allow extensive manipulation of parameters, even in scenarios that may not have immediate biological relevance or experimental basis. However, these choices could be motivated by the fact that this model serves as more than just a computational environment for the replication of colorectal cancer (CRC) development. In fact, it provides a platform for theoretical inquiries into causal relationships within a biologically inspired system. For example, one might want to set the physiological cell lifespan to just 1 h or allow physiological cells to enter mitosis activity regardless of their neighborhood. While these are not realistic scenarios, biologically speaking, they are possible within the model context. Another example is the activation or deactivation of the immune system cell response, to observe whether population-specific tendencies emerge from the interaction between neoplastic cells and immune system cells. Hence, instead of focusing only on positive results, a researcher could use the model to eliminate all biologically meaningless scenarios.

Identifying causal relationships in traditional in vivo and in vitro research can be difficult due to the complex nature of the entities and interactions involved, as well as the difficulty of performing counterfactual experiments while keeping some variables unchanged and adjusting the value of others. However, within the precisely controlled environment offered by this model, an interventionist account of causality [60,61] can be effectively applied. Within this approach, causation is understood through the potential to manipulate one variable to bring about a change in another. If changing *X* through an intervention leads to a change in *Y*, then *X* is considered a cause of *Y*. The theory relies on counterfactuals, meaning it examines what would happen to *Y* if *X* were manipulated differently. This helps to determine the causal impact of *X* on *Y*. Moreover, the manipulability of the model allows for the exploration of intrinsic and extrinsic interactions that contribute to the neoplastic phenomenon [62]. For example, the current model represents driver genes as causal agents with precise effects (intrinsic factors), without considering any reciprocal influence such as epistasis. For instance, this means that the altered movement resulting from *APC* suppression does not directly affect the proliferation time, which only increases after a *KRAS* mutation. Regarding extrinsic factors, the large population of cells in the same location influencing each other and potentially leading to cell death can be considered as an environmental influence, or constraint, shaping subsequent cell behavior within the spatial context [35,63,64].

Concerning the initial genotype frequency, all the runs were conducted with a starting probability of heterozygosity set at 0.5, meaning that every first-line cell has a 1/2 probability of being heterozygous. Therefore, it is necessary to perform tests with other starting values, such as a completely healthy population of first-line cells with wild-type genes (i.e., a probability of heterozygosity set to zero). It is also worth mentioning that the model represents an idealized crypt without specific characteristics unique to any particular human or murine individual. Therefore, aspects such as the one-year simulation period or the number of cells required to trigger the population stop condition (10,000 cells) do not reflect the characteristics of patients at specific ages or tumor stages.

Further testing is needed for parameters related to immune system cells and overcrowding deaths. For example, a minimal genetic model of tumor development can be explored by setting the number of immune cells to zero and the hypoxic threshold to a large number. In this scenario, we would expect to observe distinct tumor development once the first neoplastic cell arises. Consistent with the Vogelstein model, we observed that in all simulations where neoplastic phenotypes emerged, cells initially assumed the *adenoma* phenotype (i.e., *KRAS* mutation). This is possibly due to the fact that *KRAS* acts as an oncogene even with one mutated allele and genotype frequencies indicate that the majority of the population is heterozygous for *KRAS*.

## 5. Conclusions

In this work, we attempted to develop a model that takes into account both genetic and environmental components in colorectal cancer development. **SMT** (*somatic mutation theory*) and **TOFT** (*tissue organization field theory*) are the two main paradigms used in cancer research that represent these two aspects. The first theory posits that multiple consecutive gene mutations in a single cell, which then proliferates more rapidly than others, are the primary drivers of a tumoral phenotype’s spread and the main cause of tumor emergence and development. The second theory suggests that environmental factors cause alterations in the signaling between cellular populations in adjacent tissues, leading to gene mutations [65,66]. The connection between these two aspects, even if it is oversimplified, is built in a way that is consistent with what cancer research indicates, namely that it is not just a matter of mutation and hyperproliferation. The presence of immune system cells can favor the rise of one cell typology over another and an elevated number of cells in a small space causes cells to die. Additional research should provide more precise evidence of correlations (and hopefully causal relationships) between system variables and environmental factors that affect the dynamics of different cell typologies. Moreover, future work will aim to incorporate additional features at the genetic and cellular levels, such as energy consumption based on cell typology, a more detailed model of gene regulatory networks for driver genes, and more sophisticated interaction between crypt cells and the immune system.

## Figures and Tables

**Table 1:** Values of the main cell variables. Arrows represent the movement of the cells in the crypt and the direction where the daughter cell is placed. Details about APC, KRAS, and TP53 are presented in Table 2.

Main Cell Variables	Values
**Lifetime**	physiological = min 96 h (hours), max 120 h; Tumoral = min 96 h, max 150 h.
**Mitosis time** ^1^	Normal distribution, mean = Mitosis mean time, variance 1.
**Mitosis mean time**	physiological (*transit-amplifying*, *differentiated cells*) = 24 h; with KRAS heterozygote = 12 h; with typology *tumoral* = 10 h.
**β-cat**	Descendant gradient: 1 at the bottom, 0 at the top of the crypt.
**Neoplastic typologies**	*pre-adenoma:* true if APC = [1 1]
	*adenoma:* true if APC = [1 1] and K-ras = [1 0] or [0 1]
	*tumoral:* true if APC = [1 1], K-ras = [1 0] and P53 = [1 1]
**Movement**	Physiological cells = ↑
	*pre-adenoma* = ↖,↗
	*adenoma* = ↖,↗
	*tumoral* = no movement
**Proliferation directions**	Physiological cells = ⟷
	*pre-adenoma* = ⟷,↖,↗
	*adenoma* = ⟷,↖,↗
	*tumoral* = ⟷,↖,↗,↙,↘,↓,↑

^1^ Mitosis is the process of replication of a single cell, which produces two genetically identical cells. We opted to model the distribution of mitosis time as approximately normal, influenced by its implementation in other computational models, see [20,43]. This choice was also prompted by the limited availability of specific data on the population distribution of this feature in the literature, despite the existence of numerous studies exploring the life cycle and division timing of individual cells; see [31,37,47].

**Table 2:** Gene characteristics show when the respective function is activated, (depending on the allele status).

Genes	State
**APC**	wild-type = [0 0], heterozygote = [1 0] [0 1],
	mutated = [1 1] (trigger *pre-adenoma* typology)
**KRAS**	wild-type = [0 0], heterozygote = [1 0] [0 1]
	(trigger *adenoma* typology)
**TP53**	wild-type = [0 0], heterozygote = [1 0] [0 1],
	mutated = [1 1] (trigger *tumoral* typology)
**Regulation genes** (N = 50)	N = [ [0 0], [0 1], …, [1 0] ]
	if a given threshold *x* is passed
	and *P53* = [1 1], trigger cells death

## Data Availability

The original data presented in this study are openly available on GitHub at https://github.com/MLedda7/CRC-Model (accessed on 20 October 2024), and Zenodo at https://doi.org/10.5281/zenodo.10669907 (accessed on 16 February 2024) and https://doi.org/10.5281/zenodo.13837071 (accessed on 25 September 2024).

## Abbreviations

The following abbreviations are used in this manuscript:

CRC	colorectal cancer
SMT	somatic mutation theory
TOFT	tissue organization field theory
TME	tumor microenvironment
